# Effects of transcutaneous electrical nerve stimulation on pain, walking function, respiratory muscle strength and vital capacity in kidney donors: a protocol of a randomized controlled trial

**DOI:** 10.1186/1471-2369-14-7

**Published:** 2013-01-11

**Authors:** Thiago Tafarel Galli, Luciana Dias Chiavegato, Nathália Risso Santiago, Richard Eloin Liebano

**Affiliations:** 1 Physical Therapy Department, University of the City of Sao Paulo (UNICID), Sao Paulo, Brazil

**Keywords:** Transcutaneous electric nerve stimulation, Postoperative pain, Nephrectomy, Kidney transplantation, Respiratory function tests

## Abstract

**Background:**

Pain is a negative factor in the recovery process of postoperative patients, causing pulmonary alterations and complications and affecting functional capacity. Thus, it is plausible to introduce transcutaneous electrical nerve stimulation (TENS) for pain relief to subsequently reduce complications caused by this pain in the postoperative period. The objective of this paper is to assess the effects of TENS on pain, walking function, respiratory muscle strength and vital capacity in kidney donors.

**Methods/design:**

Seventy-four patients will be randomly allocated into 2 groups: active TENS or placebo TENS. All patients will be assessed for pain intensity, walk function (Iowa Gait Test), respiratory muscle strength (maximal inspiratory pressure and maximal expiratory pressure) and vital capacity before and after the TENS application. The data will be collected by an assessor who is blinded to the group allocation.

**Discussion:**

This study is the first to examine the effects of TENS in this population. TENS during the postoperative period may result in pain relief and improvements in pulmonary tests and mobility, thus leading to an improved quality of life and further promoting organ donation.

**Trial registration:**

Registro Brasileiro de Ensaios Clinicos (ReBEC), number RBR-8xtkjp.

## Background

Organ donation has grown exponentially in Brazil, which is directly reflected by the significant increase in the number of transplants. A successful transplant affects both the lifespan and quality of life of patients. A renal graft from a living donor provides a 10 to 12% better chance of survival at one year [1]. There are several methods used to remove a kidney, and all are designed to minimize the harm to the donor. Methods that have been used since 1995 include lumbotomy, the subcostal access route and laparoscopic access routes [2-4].

The postoperative complications in patients who underwent nephrectomy can be divided into mechanical, pulmonary, vascular and operative wounds [5]. The major pulmonary complications during this period included the following: atelectasis, pneumothorax, pulmonary congestion, pneumonia and hypoxemia [6]. Pain is another factor that negatively influences the postoperative outcomes of patients, even with the administration of analgesics, including the most widely used opioids [7]. The major difficulty when using opioids is calculating the minimum concentration for effective analgesia, as overuse can lead to cough suppression, can limit effective sputum expulsion and can cause dizziness, nausea, vomiting, sedation, urinary retention and constipation, which are known side effects of opioids [8]. For these reasons, other non-pharmacological methods of pain control can help limit the use of opioids and reduce side effects [7].

Transcutaneous electrical nerve stimulation (TENS) is considered to be a non-pharmacological intervention strategy used for physical therapy pain relief with minimal side effects [9]. The use of TENS activates opioid receptors, which reduce the activation of the nociceptive sensory pathways and provide pain relief [10-12]. TENS has been used as a therapeutic option to control chronic and acute pain since the early 1970s. Its postoperative use following cardiac and thoracic surgery provides decreased analgesic consumption, lower incidence of pulmonary complications (such as atelectasis) and improved pulmonary function [13,14]. However, the positive effects of TENS have not been consistent. Some researchers found that TENS was not effective in all patients, especially those with more severe postoperative pain. Additionally, the declines in lung function and ventilation did not always responded positively after the use of TENS. Therefore, the actual role of TENS in controlling postoperative pain and its consequences remains controversial [6,13,15].

There has been a significant increase in the rate of kidney transplants performed using living donors in recent years, raising questions regarding the short- and long-term effects of this procedure, such as whether there would there be a difference in pain intensity or changes in pulmonary function and donor independence or functionality in the postoperative period after the use of TENS. Moreover, no previous studies were found in the literature that investigated the effects of TENS in this population. For these reasons, we decided to conduct a prospective randomized controlled study to follow kidney donors who underwent lumbotomy or subcostal nephrectomy to evaluate the effect of TENS on pain, walking function, respiratory muscle strength and vital capacity.

## Methods and design

### Study design

This study is a prospective randomized controlled and double-blind. Seventy-four patients will be randomly distributed into two groups: active TENS + analgesics (n = 37) and placebo TENS + analgesics (n = 37). Randomization will be performed by an investigator not involved in data collection using the sequentially numbered, opaque sealed envelopes (SNOSE) allocation concealment method [16,17]. Patients will be admitted one day before surgery and will undergo group randomization on the first day of the postoperative period. After randomization, the patients will be assessed for pain intensity, respiratory muscle strength, vital capacity and walking function before and after the use of TENS. The present study was approved by the Research Ethics Committees of University of the City of Sao Paulo and Federal University of Sao Paulo. All participants will receive written and verbal information on the aims and procedures of the study and will sign a consent form to agree to participate in the study.

**Figure 1:**
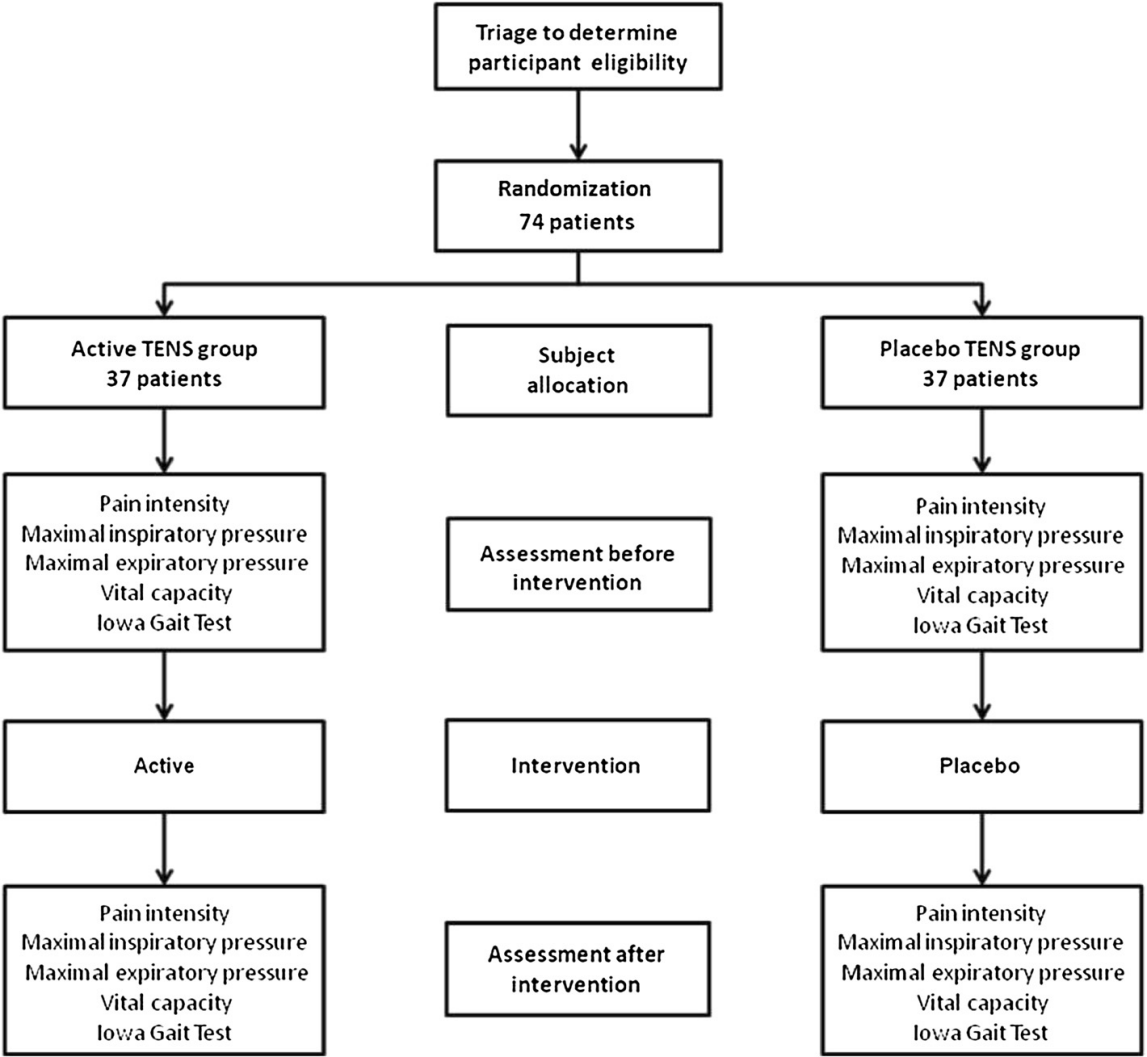
Study flow diagram of the randomized controlled trial.

All patients will be advised on the assessments that will be performed, including general information on the surgical procedure (type and size of the incision, sedation, intubation and need for admission to the Intensive Care Unit). The study flow diagram is presented in Figure 1.

### Participants

This study will be conducted in the Kidney and Hypertension Hospital - Oswaldo Ramos Foundation, a supplementary service of the Federal University of Sao Paulo. The study will include 74 living kidney donors who underwent nephrectomy performed by two approaches: lumbotomy and the subcostal route. The route choice is dependent on the team performing the procedure, and these teams operate alternately on weekdays.

### Inclusion criteria

• All living kidney donors undergoing nephrectomy by lumbotomy or the subcostal route;

• At least 18 years old and of either sex;

• Without acute or chronic pulmonary disease;

• Ability to perform the necessary physiotherapy maneuvers and measures;

• Pain intensity at rest greater than or equal to 2 and less than or equal to 7 on a numeric rating scale (0–10)

### Exclusion criteria

• All living kidney donors who have previous experience with TENS;

• Need for surgical intervention on another organ in addition to the kidney;

• Surgical reintervention during the data collection period;

• Required mechanical ventilation for more than 12 hours;

• Died within 6 hours of the operation

### Intervention

#### Application of active TENS

The use of active TENS will be conducted with a Neurodyn Portable TENS unit (IBRAMED) with two channels. The generator emits asymmetric, balanced, biphasic pulses. Two self-adhesive electrodes (VALUTRODE - 9 × 5 cm) will be placed in parallel on both sides of the incision with a 3-cm distance between them. The duration of the TENS application will be 1 hour at a frequency of 100 Hz and a pulse duration of 100 microseconds [18]. The intensity (amplitude) will be increased until the patient is able to feel a strong but comfortable tingling sensation. Patients will be asked about the intensity of the TENS every 10 minutes. In the case of sensory habituation, the amplitude will be increased until the individual again feels a strong but comfortable tingling sensation. The application will be performed on the first day of the postoperative period.

#### Application of placebo TENS

The use of the placebo TENS will be performed with an apparatus identical in appearance to the active TENS (Neurodyn Portable TENS, IBRAMED) that is specially designed for this study. The device remains active only during the first 30 seconds of application. After the initial 30 seconds, the current amplitude will gradually decrease over 15 seconds until it reaches the zero value, thereby interrupting the emission of electric current. The unit will remain inactive for the rest of the application [19]. The placebo TENS device displays a light during the entire application, indicating to the patient that the device is active. Patients will be informed that TENS may cause a slight tingling sensation or no sensation during the procedure.

They will be asked every 10 minutes about the intensity of TENS, reinforcing the idea that lack of sensation occurs in most cases due to habituation to the electric current. Patients will be observed throughout the administration of TENS [20]. The mode of application of the electrodes and the duration of the placebo TENS will be the same as described in the active TENS.

#### Outcome measures

***Primary outcome: *pain intensity**

To evaluate pain intensity, an 11-point numeric rating scale (NRS) will be used, where 0 represents no pain and 10 represents the most intense pain imaginable. The assessment will be conducted at rest, during coughing and during respiratory tests (maximal inspiratory and expiratory pressures and vital capacity) and walking function (Iowa Gait Test) before and after administration of TENS [21].

***Secondary outcomes: *respiratory muscle strength, vital capacity and walk function**

Patients will be assessed for their respiratory muscle strength (maximal inspiratory pressure (PImax), maximal expiratory pressure (PEmax)) and vital capacity (VC). PImax and PEmax will be assessed with an analog manovacuometer (MTR) with an operating range of 300 cmH_2_O (calibrated before each use) and with the patient in the seated position with relaxed arms and a disposable mouthpiece and nasal opening for the air leak.

To measure the inspiratory muscle strength (PImax), the patient will be asked to perform a maximal expiration to the level of residual volume (RV) and then perform a maximal inspiratory effort, which must be maintained for approximately one second. To measure expiratory muscle strength (PEmax), the patient will be asked to perform a maximal inspiration at the level of total lung capacity (TLC) and then perform a maximal expiratory effort, which must be maintained for approximately one second [19,22,23]. The patient will be encouraged by the evaluator during these procedures to exert his/her maximal effort. The greatest of three consecutive measurements will be used. The mouthpieces used to record respiratory muscle strength have a small opening (± 2 mm) to exclude the force of the facial muscles and make the measurements more reliable.

The vital capacity (VC) will be measured with a spirometer (Wright Mark 8) with the patient in a seated position and connected to a disposable mouthpiece and nose clip to prevent air leakage. The test starts with inspiration to TLC, followed by expiration to the RV. The greatest of three consecutive measurements will be considered [19,22,23].

Assessment of walking function will be performed using the Iowa Gait Test. Patients will be assessed on three items: walking speed, distance travelled and level of assistance while walking for 15 seconds [24]. The evaluator will walk alongside the patient and measure time using a digital timer (Oregon SL 110). The evaluator will not talk during the test to allow the patient to determine his/her own pace and will provide the necessary assistance to the patient when needed. The starting point will be marked with tape, and the patient will be instructed to walk forward as fast and safely as possible and stop when requested. The place where the patient stops will also be marked with tape, and the distance between the two points marked will be measured. Those who cannot walk independently will receive assistance. The levels of assistance will be classified as follows:

• Independent, no assistance

• Minimal assistance, one point of contact (e.g., holding an arm)

• Moderate assistance, two points of contact (e.g., holding both arms)

• Maximum assistance, three or more points of contact (e.g., holding an arm and both hands at the waist)

The saturation of peripheral oxygen (SpO_2_) will be monitored through a pulse oximeter (Nonin Onyx 9500) to ensure proper oxygen saturation during the test. If the SpO_2_ drops below 92% or 10% of the baseline, the test will be stopped.

### Procedure

#### Active TENS group

Patients in this group will receive prescription pain medication for pain control. In addition to primary care, patients will undergo TENS on the first day of the postoperative period, with an exposure time of 1 hr. Pain intensity, pulmonary tests and walking function will be evaluated before and after the intervention with TENS, immediately before the TENS is discontinued.

#### Placebo TENS group

Patients in this group will also receive prescription of analgesics for pain control. Patients will undergo the same procedures as the TENS active group, but the unit will emit electrical current only during the first 30 seconds of the application. One hour after the commencement of the intervention, patients will be subjected to the same tests as described above.

#### Blinding

Two assessors will be involved in the study and will be trained to perform the procedures in this study. Assessor 1 will be responsible for the evaluation of pulmonary tests, walking ability and assessment of pain intensity in all patients in the study. Assessor 2 will be responsible for administering TENS in all patients in the study. Only Assessor 2 will know if the patient received the active or placebo TENS. Both Assessor 1 and the patients will be blinded to the mode of application of TENS. Assessor 2 will instruct patients not to report their perceptions during the TENS administration to the assessor conducting the assessments.

#### Data analysis

The sample size was calculated considering a difference of two points in the verbal numeric pain scale between the TENS active and placebo groups and an estimated standard deviation of 3 based on data from a previous study [25]. For a significance level of 0.05 and 80% power, it was estimated that 37 participants would be required in each group (Minitab, v.15, State College, PA).

The statistical analysis will be performed on an intent-to-treat basis. The effects of the intervention on pain intensity, pulmonary function, and walking capacity, will be calculated using independent samples t tests for normally distributed data. Data not normally distributed will be compared using Mann–Whitney U tests. The level of significance will be set at *α* = .05. The data will be analysed using SPSS software (v.17; SPSS Inc; Chicago, IL) by an investigator who is blind to group allocation.

## Discussion

The use of TENS during the postoperative period has become more common in various types of surgery in recent decades [13,14,26,27]. The postoperative benefits of TENS exceed pain relief and include decreased analgesic consumption, deceased incidence of pulmonary complications (such as atelectasis) and improved pulmonary function [6,8,13,14,28]. Several authors have evaluated the postoperative effects of TENS following cardiac surgery and found that TENS reduces pain and the amount of analgesic consumption when compared to placebos or controls [8,27,29].

An improvement in respiratory muscle strength and increased pulmonary volumes and capacity, in addition to a reduction of pain after the use of low-intensity TENS was observed in the postoperative period of cardiac surgery. This study also showed that the group that underwent TENS experienced significant increases in both PImax and PEmax compared to the control group [6]. The effects of TENS on pulmonary function was also assessed in 31 patients undergoing coronary artery bypass grafting or valve replacement during the first 72 postoperative hours. Patients were randomly assigned to two groups: TENS and placebo. As a result, an improvement in the peak expiratory flow (PEF) values in the group that underwent TENS was observed; however, when both groups were compared, there were no significant differences in the forced vital capacity (FVC) and forced expiratory volume in 1 second (FEV1) values [29]. Similar results were observed in a study of 45 patients in which TENS was used as a postoperative complement to pharmacologic analgesia in patients undergoing coronary artery bypass grafting. The authors performed three spirometric measurements at 24, 48 and 72 hours after surgery and found that there were no significant improvements in the spirometric values of patients who were treated with TENS when compared with those receiving the placebo treatment [14].

The use of TENS increases the analgesic effect in patients undergoing a thoracotomy, providing an important analgesic strategy for the treatment of acute postoperative pain [24,25,28,30,31]. However, a study was conducted with 324 patients who underwent different types of chest surgery and concluded that TENS had little or no benefit after procedures associated with severe pain (posterolateral thoracotomy) [26]. Accordingly, the results after a lateral thoracotomy or sternotomy are varied; many studies advocate the efficacy of TENS in patients undergoing these surgeries, while others claim that TENS has little or no value after these procedures [26,32].

Here, we present the rationale and design for a randomized controlled trial comparing the effects of active TENS versus placebo TENS in patients undergoing nephrectomy. This is the first study to be conducted on the use of TENS in this population. The results of this study will be published when it is completed.

## Abbreviations

TENS: Transcutaneous electrical nerve stimulation;SNOSE: Sequentially numbered, opaque sealed envelopes;NRS: Numeric rating scale;PImax: Maximal inspiratory pressure;PEmax: Maximal expiratory pressure;VC: Vital capacity;RV: Residual volume;TLC: Total lung capacity;SpO2: Saturation of peripheral oxygen;PEF: Peak expiratory flow;FVC: Forced vital capacity;FEV1: Forced expiratory volume in 1 second

## Competing interests

The authors declare that they have no competing interests.

## Authors’ contributions

REL, LDC and TTG were responsible for conceiving and designing the study. REL and LDC are the study coordinators. TTG and NRS are responsible for data collection. All authors have contributed for writing and approved this manuscript.

## Pre-publication history

The pre-publication history for this paper can be accessed here:

http://www.biomedcentral.com/1471-2369/14/7/prepub
